# The natural compound *n*-butylidenephthalide derived from *Angelica sinensis* inhibits malignant brain tumor growth *in vitro* and *in vivo*^3^

**DOI:** 10.1111/j.1471-4159.2006.04151.x

**Published:** 2006-11

**Authors:** Nu-Man Tsai, Yi-Lin Chen, Chau-Chin Lee, Po-Chen Lin, Yeung-Leung Cheng, Wen-Liang Chang, Shinn-Zong Lin, Horng-Jyh Harn

**Affiliations:** * Department of Applied Life Science, Asia University Taichung, Taiwan; † Neuro-Medical Scientific Center, Buddhist Tzu Chi General Hospital Hualien, Taiwan; ‡ Department of Radiology, Buddhist Tzu Chi General Hospital Hualien, Taiwan; § Institute of Medical Sciences, Buddhist Tzu Chi General Hospital Hualien, Taiwan; †† Department of Pathology, Buddhist Tzu Chi General Hospital Hualien, Taiwan; ‡‡ Department of Emergency Medicine, Buddhist Tzu Chi General Hospital Hualien, Taiwan; ¶ Division of Thoracic Surgery, Tri-Service General Hospital, National Defense Medical Center Taipei, Taiwan; ** School of Pharmacy, National Defense Medical Center Taipei, Taiwan

**Keywords:** *Angelica sinensis*, apoptosis, glioblastoma multiformis, *n*-butylidenephthalide

## Abstract

The naturally-occurring compound, *n*-butylidenephthalide (BP), which is isolated from the chloroform extract of *Angelica sinensis* (AS-C), has been investigated with respect to the treatment of angina. In this study, we have examined the anti-tumor effects of *n*-butylidenephthalide on glioblastoma multiforme (GBM) brain tumors both *in vitro* and *in vivo*. *In vitro*, GBM cells were treated with BP, and the effects of proliferation, cell cycle and apoptosis were determined. *In vivo*, DBTRG-05MG, the human GBM tumor, and RG2, the rat GBM tumor, were injected subcutaneously or intracerebrally with BP. The effects on tumor growth were determined by tumor volumes, magnetic resonance imaging and survival rate. Here, we report on the potency of BP in suppressing growth of malignant brain tumor cells without simultaneous fibroblast cytotocixity. BP up-regulated the expression of Cyclin Kinase Inhibitor (CKI), including p21 and p27, to decrease phosphorylation of Rb proteins, and down-regulated the cell-cycle regulators, resulting in cell arrest at the G_0_/G_1_ phase for DBTRG-05MG and RG2 cells, respectively. The apoptosis-associated proteins were dramatically increased and activated by BP in DBTRG-05MG cells and RG2 cells, but RG2 cells did not express p53 protein. *In vitro* results showed that BP triggered both p53-dependent and independent pathways for apoptosis. *In vivo*, BP not only suppressed growth of subcutaneous rat and human brain tumors but also, reduced the volume of GBM tumors *in situ*, significantly prolonging survival rate. These *in vitro* and *in vivo* anti-cancer effects indicate that BP could serve as a new anti-brain tumor drug.

Glioblastoma multiforme (GBM), the extreme expression of anaplasia among the glial neoplasmas, accounts for 40% of all primary intracranial tumors (Mahaley *et al*. 1989; Graham and Lantos 2002). The diffusely invasive properties of GBM mean that total resection is almost impossible and therefore, surgery plus radiotherapy, and eventual chemotherapy, is the standard treatment (Santarius *et al*. 1997; Graham and Lantos 2002; Giese *et al*. 2003). Nonetheless, the clinical course rarely exceeds 18 months from the time of diagnosis, regardless of therapeutic intervention (Santarius *et al*. 1997; Graham and Lantos 2002).

Chemotherapy is usually reserved for recurrent tumors that have already been treated with surgery and radiotherapy, or for tumors in which surgery was only partial or infeasible and the effect of radiotherapy was limited (Blacklock *et al*. 1986; Shapiro and Green 1987). Various chemotherapy schemes have been used, with most consisting of administering drugs at high doses (Shapiro and Green 1987). The present chemotherapy regimens are limited for several reasons, including development of toxicity and drug resistance, limited success in overcoming the blood–brain barrier (BBB) and limited therapeutics (Blacklock *et al*. 1986; Shapiro and Green 1987; Elliott *et al*. 1996; Bello *et al*. 2001; Cheng *et al*. 2004).

*Angelica sinensis* (Oliv.) Diels (AS; dong quai; also called danggui), a traditional Chinese medicine for menopausal symptoms (Ye *et al.*, 2001a; Ye *et al.*, 2001b; Yim *et al*. 2002), has been clinically administrated for several gynecological symptoms in the US (Abebe 2002). In our previous study, the chloroform extract of *A. sinensis* (AS-C) also showed a dramatic anti-tumor effect, causing growth resting and apoptosis of malignant brain tumors *in vitro* and *in vivo*, and both p53-dependent and -independent pathways of apoptosis were involved in the cytotoxic mechanisms (Tsai *et al*. 2005). Thus far six major compounds have been isolated form *A. sinensis*: (E)-liguistilide, (Z)-ligustilide, (Z)-*n*-butylidenephthalide, palmitic acid, beta-sitosterol and ferulic acid (Wang *et al*. 1998). *n*-Butylidenephthalide (BP, BdPh or K1; molecular weight approximately 188.22) derived from AS-C was the major component (over 30% of AS-C crude) and was therefore chosen in this further study to verify its anti-tumor activity.

Several medicinal properties of BP, such as the anti-platelet effect, are due mainly to an inhibitory effect on cyclo-oxygenase (Teng *et al*. 1987). It also provides relief from angina (Ko *et al*. 1998, 2002). In addition, it has been suggested that the anti-proliferation effect of synthetic BP-42 (3-butylidene-4,5-dihydroxyphthalide), which was modified by adding the hydroxyl molecules in the phthalide group of BP, could be used as an anti-atherosclerotic treatment (Mimura *et al*. 1995). However, the anti-tumor activities of BP have not yet been determined. In this study, the anti-tumor effects of BP on malignant brain tumors *in vitro* and *in vivo* were investigated. The results revealed that the significant therapeutic anti-tumor efficacy of BP on GBM tumors involved the induction of cell-cycle rest and apoptosis.

## Materials and methods

### Chemicals

*n*-Butylidenephthalide (BP; molecular weight: 188.23), purchased from Lancaster Synthesis Ltd (Newgate, Morecambe, UK) and carmustine (BCNU; Sigma Chemical Co., St Louis, MO, USA) were dissolved in dimethylsulfoxide (DMSO) and ethanol, incubated with shaking at 25°C for 1 h, and stored at 4°C before each *in vitro* experiment.

### Cell lines and cell culture

The DBTRG-05MG line of human GBM cells, RG2 line of rat GBM cells, SK-N-AS line of human neuroblastoma cells, SVEC line of mouse vascular endothelial cells and Balb/3T3 line of mouse fibroblast cells were obtained from the American Type Culture Collection (Rockville, MD, USA). G5T/VGH human GBM cells, GBM 8401 human GBM cells, GBM 8901 human GBM cells, A549 human lung adenocarcinoma cells, PA-1 human teratoma cells, B16/F10 mouse melanoma cells, HL-60 human leukemia cells and N18 mouse neuroblastoma cells were obtained from the Bioresources Collection and Research Center (BCRC, Hsin Chu, Taiwan). The J5 line of human hepatocellular carcinoma cells and BCM-1 line of human breast cancer cells were kindly provided by Drs M. J. Chou and C. S. Yang (Sherr and Robert 1995) and Dr D. S. Yu (Reed *et al*. 1994; Dobashi *et al*. 2003), respectively. The DBTRG-05MG, GBM8401, GBM8901, BCM-1, HL-60, A549, PA-1 and J5 cells were maintained in RPMI-1640 (Sigma) medium with 10% fetal bovine serum, at 37°C in a humidified atmosphere containing 5% CO_2_. The G5T/VGH, RG2, SK-N-AS, N18, B16/F10, SVEC and Balb/3T3 cells were cultured in Dulbecco's modified Eagle's medium with 10% fetal bovine serum at 37°C in a humidified atmosphere containing 5% CO_2_.

### Cytotoxicity analysis

Viable cells were evaluated by a modified 3-(4,5-dimethylthiazol-2-yl)-2,5-diphenyltetrazolium bromide (MTT) assay. Briefly, 5 × 10^3^ cells were plated in each well of a 96-well plate, incubated overnight with 100 µL growth medium in 96-well plates, then treated with 100 µL BP dissolved in the medium (0–250 µg/mL). The concentration of DMSO was ≤ 0.02% in each preparation. After 24, 48 or 72 h of BP treatment, the medium was replaced with 50 µL fresh medium containing MTT (400 µg/mL) for 6–8 h. The MTT medium was then removed and 100 µL DMSO added to each well. The solutions were detected using an MRX Microtiter Plate Luminometer (Dynax Technologies, Chantilly, VA, USA) at 550 nm. Absorbance of untreated cells was considered as 100%. The 50% inhibitory concentration (*IC*_50_) was the concentration that caused a 50% decrease in the optical density of drug-treated cells with respect to untreated cells.

### Analysis of cell-cycle distribution

The GBM tumor cell lines DBTRG-05MG and RG2 were cultured with drug diluent for 48 h; DMSO content was controlled at ≤ 0.02% and BP at 75 µg/mL. Cell-cycle analysis was performed by DNA staining with propidium iodide (PI) and flow cytometry. Briefly, cells were harvested, resuspended in 0.8 mL 1 × phosphate-buffered saline (PBS) and added to 200 µL PI solution (50 µg/mL PI + 0.05 mg/mL RNase A; Sigma Chemical Co.). After overnight incubation at 4°C, cells were kept at 25°C for 2 h. The cells were then passed through FACScan (Becton Dickinson Immunocytometry Systems, San Jose, CA, USA) to measure the DNA content.

### Determination of apoptosis

Apoptosis was assayed using an *in Situ* Cell Death Detection Kit, POD (Roche, Mannheim, Germany), according to the manufacturer's instructions. Briefly, DBTRG-05MG and RG2 cells were cultured in culture dishes and analyzed at the indicated time points (24, 48, 72 h) after BP (75 µg/mL) treatment. In the BP-treated cell group, suspended cells were collected; in the control group, adherent cells were collected. Cells were fixed at 25°C for 15 min with 3.7% formaldehyde, smeared on silane-coated glass slides (Muto Pure Chemicals, Tokyo, Japan), washed once in 1 × PBS and incubated in cold permeabilization solution (0.1% Triton X-100 + 0.1% sodium citrate) after reducing endogenous peroxidase enzyme activity with 3% H_2_O_2_. Then, cells were incubated with terminal deoxynucleotidyl transferase (TdT)-mediated dUTP nick end labeling (TUNEL) reaction mixture for 60 min at 37°C, and counterstained with PI for determination of the cell count. To quantify cell apoptosis, the slides were viewed under fluorescence microscopy (Nikon, Kawasaki, Japan).

### Western blot analysis

DBTRG-05MG and RG2 cells were seeded in a six-well plate and later treated with BP (75 µg/mL) for different times (0, 1.5, 3, 6, 12, 24 or 48 h; 0 h as a vehicle control). Cell pellets were resuspended in lysis buffer [10 nm Tris-HCl (pH 7.5), 1 mm EGTA, 0.5% 3-[3-(cholamidopropyl)dimethylammonio]-1-propanesulfonate, 10% (v/v) glycerol, 5 mmβ-2-mercaptoethanol and 0.1 mm phenylmethylsulfonyl fluoride] and incubated on ice for 30 min. After centrifugation at 13 000 r.p.m. for 20 min at 4°C, total cell lysates were collected. The protein concentration of the cell lysates was measured with a bicinchoninic acid (BCA) protein assay kit (Pierce, Rockford, IL, USA) following the manufacturer's instructions. Aliquots (20 µg) of the cell lysates were separated by 10–12% sodium dodecyl sulfate–polyacrylamide gel electrophoresis (SDS–PAGE; Bio-Rad, Hercules, CA, USA) and transferred to polyvinylidenedifluoride (PVDF) membranes (Amersham Lifesciences, Piscataway, NJ, USA). The membranes were blocked with 5% skim milk for 1 h at 25°C, then incubated with the respective antibodies. The antibodies included anti-Fas, anti-Fas-L, anti-caspase 3, anti-caspase 8, anti-caspase 9, anti-Bax, anti-AIF, anti-p16, anti-p21, anti-p27, anti-p53, anti-cdk2, anti-cdk4, anti-cdk6, anti-cyclin D1, anti-cyclin E, anti-actin (1/200 dilution; Santa Cruz Biotechnology Inc., Santa Cruz, CA, USA); anti-phospho-p53 (Ser15; 1/2000 dilution), and anti-phospho-RB (Ser795; 1/2000 dilution; Cell Signaling Technology, Beverly, MA, USA).The immobilized primary antigen-antibody complex was detected with the respective horseradish peroxidase-conjugated anti-mouse, anti-rabbit or anti-goat IgG secondary antibodies (1/1000 dilution; Santa Cruz Biotechnology Inc.) for 1 h at 25°C, then visualized with an enhanced chemiluminescence (ECL) Plus chemiluminescence system (Amersham, Arlington Heights, IL, USA). The degree of protein expression was calculated as the expression index. The expression indexes were calculated as [(sample intensity/sample β-actin intensity)/(vehicle control intensity/vehicle control β-actin intensity)].

### Animal studies

To examine the anti-tumor effects of BP *in vivo*, RG2 rat GBM cells were used in male F344 rat (230–260 g) experiments, and DBTRG-05MG human GBM cells were used in male Foxn1 nu/nu mouse (10–12 weeks) experiments. The rats and mice were purchased from the National Laboratory Animal Center (Taipei, Taiwan). All procedures were performed in compliance with the standard operating procedures of the Laboratory Animal Center of Tzu Chi University (Hualien, Taiwan).

*In vitro* cultured RG2 cells (1 × 10^6^) were injected s.c. into the back of syngeneic F344 rats. Six animals in each vehicle or BP group were treated on days 3, 6 and 9 after tumor cell implantation by s.c. injection of BP (500 mg/kg/day) or vehicle (10 mg/mL Tween-80 and 50 mg/mL propylene glycol in distilled water; Standard Chem. & Pharm., Tainan, Taiwan), far from the inoculated tumor cell sites (> 1.5 cm). In addition, DBTRG-05MG cells (2.5 × 10^6^) were injected s.c. into the backs of nude mice (five to six/group), and treated with vehicle, BP−70, −150, −300, −500 and −800 mg/kg/day (s.c.) respectively. The injection site was far from the inoculated tumor sites (>1.5 cm away from treated site), on days 4 , 5, 6, 7 and 8 after tumor cell implantation. Tumor size was measured with calipers and the volume was calculated as L × H × W × 0.5236 (Bardon *et al*. 2002). Animals were killed when the tumor volume exceeded 25 cm^3^ in rats and 1500 mm^3^ in mice; the day on which an animal was killed was designated as the final survival day for the rats and mice.

The anti-tumor effects of BP on *in situ* tumors were determined with RG2 cells. The RG2 cells were injected i.c. into the striatum of syngenic rats (six/group) and treated with BP (300 mg/kg/day) or vehicle s.c. on days 4, 5, 6, 7 and 8 after tumor cell implantation. Tumor volume was measured using 3-T unit magnetic resonance imaging (MRI; General Electric, Milwaukee, Wisconsin, USA) with echo-planar imaging capability (Fukuoka *et al*. 2000) (Signa LX 3.8; General Electric) in Buddhist Tzu Chi General Hospital (Hualien, Taiwan). Briefly, rats were anesthetized with chloral hydrate (400 mg/mL, 1 mL/100 g). Functional MRI scanning was conducted with a fast spin echo and an echo-planar acquisition sequence in which the repetition time was 6000 ms, the echo time 102 ms, the matrix image 256 × 256 pixels, the field of view 5 × 5 cm and the in-plane resolution, 80 µm. Twenty slices, each 1.5 mm thick, were obtained every 19.5 s for a total time of 6.5 min/rat.

### Immunohistochemical staining

All tumor tissues (s.c. or i.c. GBM tumors with or without BP treatments) were harvested and fixed. Paraffin-embedded sections (7 µm/section) were obtained from the tumors and processed for immunohistochemical staining. A goat polyclonal anti-Ki-67 antibody (M-19; 1/100 dilution; Santa Cruz Biotechnology Inc.) and a rabbit polyclonal anti-cleaved caspase 3 (Asp175) antibody (1/1000 dilution; Cell Signaling Technology.) were used in immunohistochemical studies with 4°C overnight incubation. The immune complexes were visualized using the horseradish peroxidase-conjugated anti-goat IgG secondary antibodies (1/1000 dilution; Santa Cruz Biotechnology Inc.) and LSAB2 system (DAKO, Corp., Carpinteria, CA, USA), respectively, and then incubated for 10 min with 0.5 mg/mL diaminobenzidine and 0.03% (v/v) H_2_O_2_ in PBS. Finally, sections were counterstained with hematoxylin, mounted, observed under a light microscope at magnifications of 400×, and photographed.

### Statistics

Data are presented as mean ± SD or SE (standard deviation and standard error, respectively). Statistical significance was analyzed by Student's *t*-test. The survival analysis was performed using the Kaplan–Meier method. A *p*-value of < 0.05 was considered to be statistically significant.

## Results

### Cytotoxic effects of BP on tumor and other cell lines

The anti-proliferative effects of BP on cells from GBM, neuroblastoma, lung cancer, melanoma, teratoma, leukemia, breast cancer and hepatocellular carcinoma, as well as from normal fibroblast and vascular endothelial cells, were determined. The *IC*_50_ values of BP after a 48 h incubation with tumor cell lines (*IC*_50_: 15–67 µg/mL) were significantly lower than the values for normal fibroblast cells (*IC*_50_: > 100 µg/mL, *p* < 0.0001) or carmustine (BCNU)-treated tumor cells (*IC*_50_: 40 ∼ 100 µg/mL, *p* < 0.001; Table 1). In the two normal cell types, vascular endothelial cells (*IC*_50_ = 25.0 ± 2.0 µg/mL) were more sensitive to BP than fibroblast cells (*IC*_50_ > 100 µg/mL) (*p <* 0.0001). Briefly, these experiments showed that BP could induce high cytotoxicity against brain and other tumor cells but only low, or no cytotoxicity against normal fibroblast cells.

**Table 1:** The *IC*_50_s of BP in different tumors 
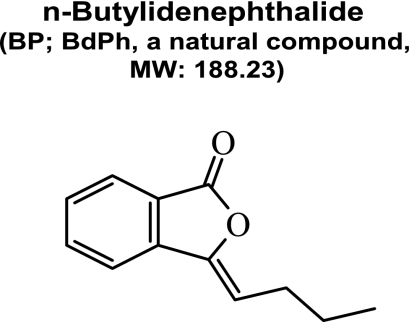

Cell line	Tumor type	BP	Carmustine
Brain tumors			
DBTRG-05MG	Human GBM cell	39.1 ± 1.7*	>100
GBM8401	Human GBM cell	21.1 ±1.5*	55.6 ± 9.6
GBM8901	Human GBM cell	28.9 ± 1.0*	56.5 ± 1.7
G5T/VGH	Human GBM cell	23.9 ± 0.9*	>100
RG2	Rat GBM cell	46.0 ± 2.7*	>100
SK-N-AS	Human neuroblastoma cell	67.4 ± 3.1*	>100
N18	Mouse neuroblastoma cell	15.5 ± 2.5*	>100
Other tumors			
A549	Human lung cancer cell	32.1 ± 0.9*	>100
B16/F10	Mouse melanoma cell	24.3 ± 1.3*	69.9 ± 1.0
J5	Human hepatoma cell	19.5 ± 0.4*	49.2 ± 0.8
PA-1	Human teratoma cell	18.7 ±1.9*	54.7 ± 9.4
BCM-1	Human breast cancer cell	50.4 ± 0.7*	>100
HL-60	Human leukemia cell	26.6 ± 8.6*	40.2 ± 9.6
Normal cells			
SVEC	Mouse vascular endothelia cell	25.0 ± 2.0†	34.5 ± 4.3
Balb/3T3	Mouse fibroblast cell	>100	>100

Note: Values are mean ± SD *IC*_50_ (μg/mL) in day 2.

* Significant difference from the BP versus Carmustine treatment in tumor cells (*p* < 0.001).

† Significant difference from SVEC versus Balb/3T3 treatment with BP (*p* < 0.0001).

**Fig. 1:**
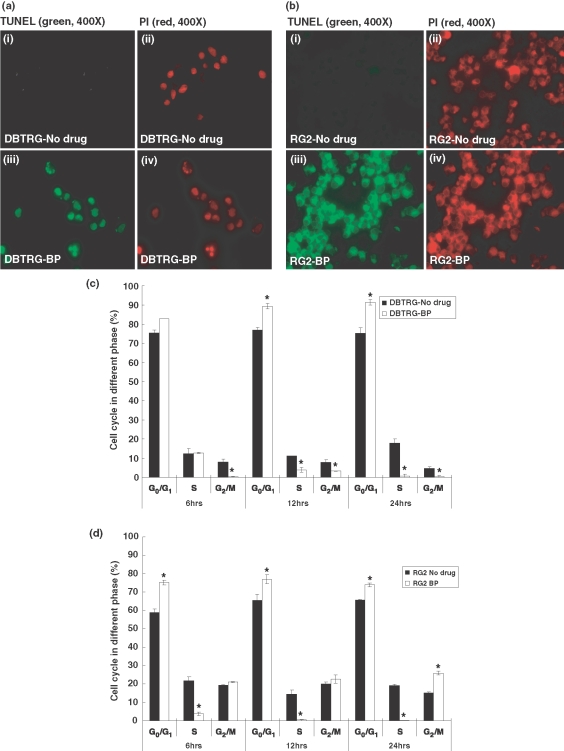
BP-induced cell-cycle arrest and apoptosis in GBM cells. DBTRG-05MG and RG2 cells underwent apoptotic cell death after a 48 h treatment with 75 µg/mL BP (a and b), as determined by TUNEL assay for DNA fragmentation (iii and vii) and PI counterstaining for genomic DNA (iv and viii). Control cells were trypsinized and stained in parallel with BP-treated cells (i, ii, v and vi). (c and d) DBTRG-05MG and RG2 cells were arrested in the G_0_/G_1_ phase with 75 µg/mL BP. Flow cytometric analysis of DNA contents in the BP-treated (open bars) and control cells (black bars) revealed the proportions of cells at different cell-cycle stages after BP treatment for 6, 12 and 24 h. Each column represents the mean ± SD (*p < 0.05).

The morphology of GBM tumor cells gradually changed with detachment from the bottom of the culture plates, after BP treatment (data not shown). The TUNEL assay was used to verify the viability of detached cells and the results showed that the cells were in the process of apoptosis at 48 h (Figs 1a and b). Therefore, BP has a cytotoxic ability against tumor cells.

### The effects of BP on cell cycle of GBM cells

Cell-cycle analysis with GBM cells demonstrated that 75 µg/mL BP treatment resulted in cell-cycle arrest at the G_0_/G_1_ phase (> 90% and > 70%; Figs 1c and d). BP induced a significant proportion of cells to arrest at the G_0_/G_1_ phase, accompanied by a concurrent decrease of S phase from 12 to 24 h (*p* < 0.05). In addition, BP also led to a decrease in the proportion of the DBTRG-05MG cells that entered the G_2_/M phase, though more cells accumulated at G_2_/M phase in RG2 cells. The results at 48 and 72 h were similar to those at 24 h (data not shown).

### Apoptotic pathways in GBM cells induced by BP

**Fig. 2:**
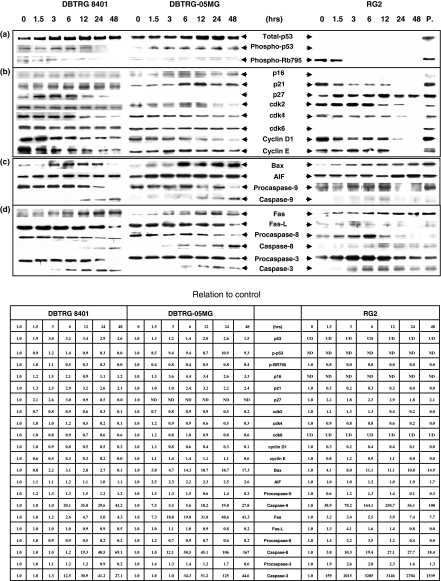
The expression and activation of apoptosis-associated molecules in GBM cells treated with BP. Whole cell lysates (20 µg/lane) were analyzed with western blotting using specific antibodies to: (a) p53, phospho-p53 and phospho-RB; (b) p16, p21, p27, cdk2, cdk4, cdk6, cyclin D1 and cyclin E; (c) Bax, AIF and caspase 9; (d) Fas, Fas-L, caspase 8 and caspase 3. Lower panel: Relation to control in (a) to (d) is relative to untreated control cells. Positive control of RG2 cells is DBTRG-05 MG cells with BP treatment for 3 h. UD, protein undetectable in this western blot system: ND, not detected.

To investigate apoptotic pathways induced by BP treatment, phosphorylation of p53 and Rb proteins were first evaluated by immunoblotting. The DBTRG-05MG and DBTRG 8401 line of human GBM cells, and RG2 line of rat GBM cells, were used for comparison. BP caused levels of phosphorylated p53 protein to increase (9.4-fold) 3 h after treatment (Fig. 2a). Furthermore, total p53 protein increased (twofold) at 12 h in DBTRG-05MG and DBTRG 8401 (twofold) increased at 3 h. In RG2 cells, the genes of p53 and p16 are impaired and exhibit homologous deletion, and these were undetectable in immunoblotting analysis. However, levels of phosphorylated Rb proteins were decreased after BP treatment in DBTRG-05MG and DBTRG 8401 cells; in RG2 cells, the levels were decreased (0.8-fold) at 1.5 h, with phosphorylated proteins undetectable as early as 3 h after BP treatment (Fig. 2a). These results indicate that BP can trigger cell-cycle checkpoint machinery. Levels of p16, p21, Bax and AIF protein in BP-treated GBM cells were therefore measured, and protein expression levels of all four proteins increased in DBTRG-05MG and DBTRG 8401 cells; similar results for p27, Bax and AIF were detected in RG2 cells after treatment with BP (Figs 2b and c). The protein expression of cdk2, cdk4, cdk6, cyclin D_1_ and cyclin E in BP-treated GBM cells was also measured, and all five proteins were decreased in DBTRG-05MG and DBTRG 8401 cells; similar results for cdk2, cdk4, cdk6, cyclin D_1_, cyclin E and p21 were detected in RG2 cells after treatment with BP (Fig. 2b).

Expression of the proteins Fas and Fas-L in treated DBTRG-05MG, DBTRG 8401 and RG2 cells was studied. Results showed that BP significantly induced Fas expression (from one to 41.3-fold vs. one to 7.7-fold in DBTRG-05MG and RG2 cells, respectively) but not Fas-L expression. In addition, activation of death receptor-induced apoptosis-related caspase 8 protein was monitored. Levels of caspase 8 in DBTRG-05MG and DBTRG 8401 were increased in a dose-dependent manner from 6 to 48 h after BP treatment, whereas the maximum expression of caspase 8 in RG2 cells at 24 h (Fig. 2d).

Finally, activation of pro-caspase 9 and procaspase 3 was also determined (Figs 2c and d). Both procaspase 9 and procaspase 3 were highly activated in DBTRG-05MG, DBTRG 8401 and RG2 cells after BP treatment.

### Anti-tumor effects of BP on survival of animals bearing subcutaneous GBM tumor

**Fig. 3:**
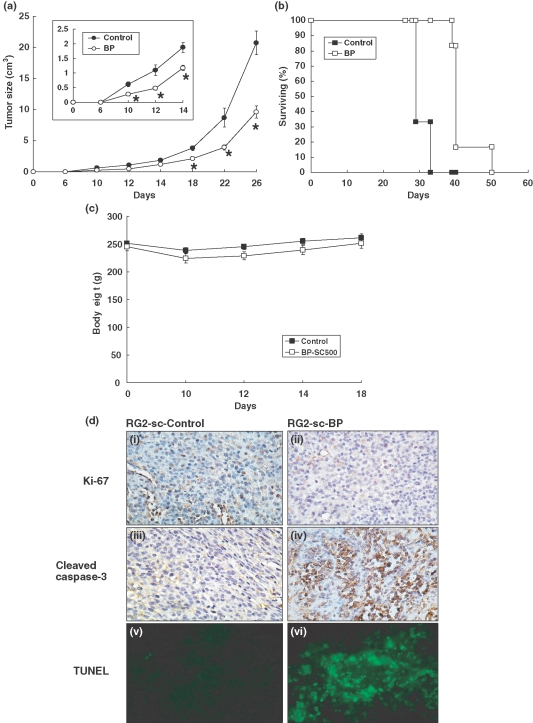
BP inhibition of tumor growth with improved survival rate in a syngenic rat GBM model. RG2 cells (1 × 10^6^) were implanted s.c. into the hind flank region of F344 rats. (a) Tumor size was measured using calipers (*p < 0.05). (b) Survival was monitored daily (*p* < 0.0001). Rats were killed when tumor size exceeded 25 cm^3^. Tumor sizes are represented as means ± SEM. There was no statistically significant difference between the control and BP-treated groups with respect to the body weight of rats. (c) Body weights are represented as means ± SEM. (d) Immunohistochemical staining and TUNEL assay were analyzed in GBM tumor tissues (at day 18 after treatment). Representative photographs of sections of the control group (i, iii and v) and BP-treated group (ii, iv and vi) GBM tumors, immunohistochemically stained for cell proliferation marker with Ki-67 (i and ii), cell apoptosis marker with cleaved caspase 3 (iii and iv) and DNA fragmentation of apoptosis cells with TUNEL staining (v and vi). The Ki-67- and caspase 3-positive cells were stained brown and the TUNEL-positive cells were stained green (×400).

To investigate BP anti-tumor activity, *in vivo* animal experiments were carried out. There was a significantly higher inhibitory effect on RG2 tumor growth in the BP-treated group than in the control (vehicle only) group (Fig. 3a; *p* < 0.005). The average tumor size at day 26 was 20.7 ± 1.5 cm^3^ for the control group, in contrast to a size of only 9.6 ± 0.4 cm^3^ for the BP-treated group. Survival of rats in the BP treatment group was significantly prolonged compared with survival in the control group (30 ± 2.1 days vs. 41.5 ± 4.2 days; Fig. 3b; *p* < 0.0001). There were no significant differences between the control and BP-treated groups with respect to the body weight of the rats after BP treatment (Fig. 3c). There were remarkable decreases in Ki-67 (a cell proliferation marker) protein expression in this study, which indicated that BP had anti-proliferation activity *in vivo*. Cleaved caspase 3 is an apoptotic marker, and the results showed that BP treatment increased the cleavage of caspase 3 protein to induce apoptosis of tumor cells *in vivo* (Fig. 3d). Both the inhibition of tumor cell growth and the induction of tumor cell apoptosis pathways were mediated by BP treatment *in vitro* and *in vivo*. With s.c. injection of BP at a dose of 500 mg/kg, no drug-related toxicity was observed based on the histological analysis of organs in animals treated with BP (data not shown).

### Anti-tumor effects of BP on *in situ* GBM tumor of rats

**Fig. 4:**
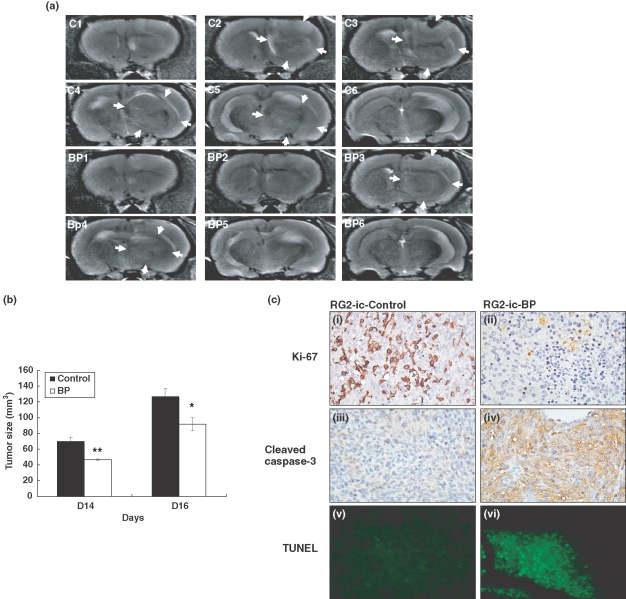
BP reduction in tumor volume in a syngenic rat GBM tumor *in situ* model. RG2 cells (5 × 10^4^) were implanted i.c. (striatum) in F344 rats. (a) Tumor volume shown by MRI imaging of serial sections (1.5 μm thick): C1–C6 were from the vehicle control rat; BP1–BP6 from a BP-treated rat (tumor mass shown by white arrow). (b) Tumor volume was calculated using echo-planar imaging capability. Each column represents mean ± SEM (*p < 0.05, **p < 0.001). (c) Immunohistochemical staining and TUNEL assay were performed in rat brain tumor tissues (on day 16 after BP treatment). Representative photographs of sections of the control group (i, iii and v) and BP-treated group (ii, iv and vi) GBM tumors immunohistochemically stained for cell proliferation marker with Ki-67 (i and ii), cell apoptosis marker with caspase 3 (active form; iii and iv) and DNA fragmentation of apoptosis cell with TUNEL staining (v and vi). The Ki-67- and caspase 3-positive cells were stained brown and the TUNEL-positive cells were stained green (×400).

To verify the BP anti-tumor effects on the *in situ* GBM tumors in rats, F344 rats were implanted i.c. (striatum) with 5 × 10^4^ RG2 cells and treated with s.c. BP (300 mg/kg/day) on days 4, 5, 6, 7 and 8. MRI data revealed that the *in situ* tumor volumes in the BP-treated group were smaller than those in the control group (Fig. 4a). There were significant declines in tumor volume for the treated group relative to the untreated group (Fig. 4b; *p* < 0.05). The mean tumor volumes at days 14 and 16 were 70.0 ± 4.8 mm^3^ and 126.4 ± 11.1 mm^3^ for the control group vs. 46.6 ± 1.8 mm^3^ and 91.7 ± 8.3 mm^3^ for the BP-treated group. The immunohistochemistry results on day 16 after BP treatment showed a decrease in Ki-67 protein expression, an increase in cleaved caspase 3 protein expression and an increase in apoptosis in tumor cells relative to the control group *in vivo* (Fig. 4c); these results are similar to those shown in the subcutaneous RG2 tumor model. Survival was significantly prolonged for the BP-treated rats compared with rats of the control group. At day 19 after BP treatment, BP caused higher survival rates (50%, 3/6) than the untreated group (16.7%, 1/6) (*p* = 0.0016; data not shown).

### Anti-tumor effects of BP on xenograft tumor growth

**Fig. 5:**
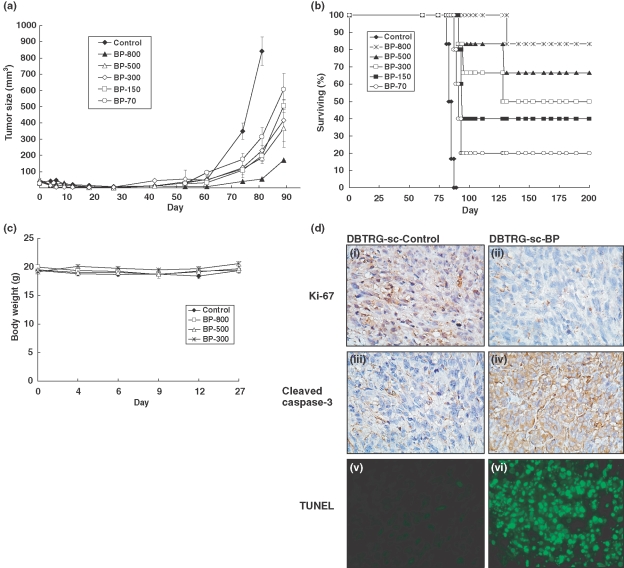
BP suppression of xenograft tumor growth of human GBM in nude mice. (a) DBTRG-05MG cells (2.5 × 10^6^) were implanted s.c. into the hind flank region of Foxn1 nu/nu mice. These mice were treated with vehicle as a control (*n* = 6), BP-70 (70 mg/kg, *n* = 5), BP-150 (150 mg/kg, *n* = 5), BP-300 (300 mg/kg, *n* = 6), BP-500 (500 mg/kg, *n* = 6) and BP-800 (800 mg/kg, *n* = 6) s.c. on days 4, 5, 6, 7 and 8. The tumor sizes were measured using calipers. There was a significant difference between the BP-treated group and the control group with respect to the tumor size (*p* < 0.05). Tumor sizes are presented as mean ± SEM. (b) The survival of mice was monitored daily (*p* < 0.001). Mice were killed when the tumor size exceeded 1000 mm^3^. (c) The body weights of mice in the control group, and in the BP-300-, BP-500- and BP-800-treated groups, were not significantly different after BP treatment; body weights are presented as means ± SEM. (d) Immunohistochemical staining and TUNEL assay were performed in human GBM tumor tissues (on day 10 after treatment). Representative photographs of sections of the control group (i, iii and v) and BP-treated group (ii, iv and vi) GBM tumors were immunohistochemically stained for the cell proliferation marker, Ki-67 (i and ii), cell apoptosis marker, caspase 3 (active form; iii and iv), and DNA fragmentation of apoptosis cell with TUNEL staining (v and vi). The Ki-67- and caspase 3-positive cells were stained brown and TUNEL-positive cells were stained green (×400).

To determine whether BP can suppress human GBM tumor growth, nude mice were inoculated with human DBTRG-05MG cells and treated with BP (70, 150, 300, 500 and 800 mg/kg in the BP-70, BP-50, BP-300, BP-500 and BP-800 groups, respectively.) on days 4, 5, 6, 7 and 8. Significant suppression of tumor growth with respect to the untreated group was observed in the BP-70-, BP-150-, BP-300-, BP-500- and BP-800-treated groups. At day 200, the tumor growth rates were 100% (6/6), 80% (4/5), 80% (4/5), 50% (3/6), 33.3% (2/6) and 16.7% (1/6) in the untreated, BP-70, BP-150-, BP-300-, BP-500- and BP-800-treated groups, respectively (*p* < 0.05). The mean tumor sizes at day 89 were > 1000 mm^3^ in the control group, 605.8 ± 98.8 mm^3^ in the BP-70-treated group, 504.4 ± 38.9 mm^3^ in the BP-150-treated group, 415 ± 130 mm^3^ in the BP-300-treated group, 365.8 ± 116.7 mm^3^ in the BP-500-treated group and 171.6 mm^3^ in the BP-800-treated group (Fig. 5a; *p* < 0.05). Survival was significantly prolonged for nude mice in the BP-treated with respect to the control group (Fig. 5b;*p* < 0.001). At day 200, survival rates were 0% (0/6), 20% (1/5), 40% (2/5), 50% (3/6), 66.7% (4/6) and 83.3% (5/6) in the untreated, BP-70-, BP-150-, BP-300-, BP-500- and BP-800-treated groups, respectively. After tumor inoculation, tumor masses (average volume = 41.84 ± 4.45 mm^3^) were developed on the backs of every mouse, but the tumors of mice with BP treatment shrank or completely regressed to be undetectable on day 150. There were no significant differences between the control and BP-treated groups with respect to the body weight of the animals (Fig. 5c). Even with a high BP dose of 800 mg/kg for 5 days of serial treatment, very low or no drug-related toxicities were observed in the animals, as evaluated by histological analyses of various organs (data not shown). The human GBM tumor tissues with BP treatment *in vivo* also displayed decreases in Ki-67 expression, and increases in cleaved caspase 3 protein expression and tumor cell apoptosis, relative to the control group *in vivo*, at day 10 after treatment (Fig. 5d).

## Discussion

*n*-Butylidenephthalide (BP) contributes to a range of biological activities, including diminution of angina, platelet aggregation, proliferation, non-specific spasmosis (Ko 1980; Teng *et al*. 1987; Mimura *et al*. 1995; Ko *et al*. 1998; 2002). In this study, we demonstrated that BP also acts strongly against GBM tumors *in vitro* and *in vivo*. This anti-tumor effect is encouraging, as very few drugs currently show efficacy against malignant brain tumors.

To explore what mechanism might account for the effects of BP on GBM tumor cells, the cell cycles were monitored. Our results are entirely consistent with an inhibition of tumor growth by BP due to cell-cycle arrest at the G_0_/G_1_ phase and the induction of apoptosis (Fig. 1). The pattern of increased and decreased protein expression during cell-cycle arrest is best explained by the BP-mediated regulation of gene expression involving the cyclin/CDK/CKI cell cycle regulatory system (Figs 2a and b). The p53 molecule is a negative regulator of cell division. In response to DNA damage, oncogenic activation of other proteins and other stresses, p53 levels rise and prevent cells from entering the S phase of the cell cycle (Reed *et al*. 1994; Sherr and Robert 1995). The p16 and p21 proteins are cdk inhibitors that negatively regulate cdk or cyclin/cdk complexes (Sherr *et al*. 1995). The p16 protein, a member of the INK4 family, binds to cdk4 or cdk6 to block the kinase activities at the mid-G_1_ phase (Reed *et al*. 1994). The p21 protein binds to the cyclin/cdk complex, resulting in the inhibition of the G_1_ to S phase transition (Dobashi *et al*. 2003) via the abrogation of Rb phosphorylation by the cyclin/cdk complex. Therefore, the decrease in phosphorylated Rb should be due to a BP-triggered expression of the cdk inhibitors, which decreases the activities of the cyclin/cdk complex. A precedent for this mechanism exists with monoterpene, an essential oil of plants (like BP) that is a natural anti-cancer compound, which causes G_1_ arrest and leads to an increase in p21 expression (Bardon *et al*. 2002). Another example is aragusterol A. This compound, isolated from marine sponges, is a potent anti-cancer marine steroid that causes G_1_ arrest by down-regulation of Rb phosphorylation (Fukuoka *et al*. 2000).

The immunoblotting results inferred the possible mechanisms of apoptosis induced by BP in GBM tumor cells. First, the p53-dependent apoptosis pathway was supposed because BP treatment could promote the phosphorylation of p53 in the DBTRG tumor cells and increase p53 expression, which peaked at 12 h (Fig. 2a). Phosphorylation at the N-terminal region of p53 abolishes Mdm2 inhibition, which causes an increase in Bax transcription (Fig. 2c shows that Bax proteins were increased) and an inhibition of Bcl-2 transcription. Because of Bax/Bcl-2 imbalance, Bax can form an active homodimer to trigger cleavage of procaspase 9 (Fig. 2c shows that caspase 9 was increased) and sequentially results in procaspase 3 activation (Fig. 2d also shows that caspase 3 was increased) to proceed apoptosis processes. Second, the Fas–FasL-induced apoptosis pathway may also be involved in BP-induced apoptosis. After BP treatment, increasing expression of Fas was observed (Fig. 2d). Fas is the death receptor responsible for signaling from the cell membrane, which triggers the activation of procaspase 8 (Fig. 2d shows that caspase 8 was increased) and subsequently, promotes procaspase 3 activation resulting in apoptosis. In order to examine these parameter cause related, different pharmcological blockers had been approach. Briefly, we pre-treated DBTRG cells with the Fas/FasL antagonist, 341291 (Calbiochem), or the Bax-inhibiting peptide, 196810 (Calbiochem), then treated the cells with BP to determine the effects of these inhibitors. We found that neither Fas/Fas L nor Bax-inhibiting peptide could reverse BP-induced growth inhibition. However, BP-induced growth inhibition effect might be attenuated by the MGMT (O^6^-methylguanine-DNA methyltransferase) tranfection assay (data not shown). MGMT is a recognized DNA repair gene (Tano *et al*. 1997). These results suggest that BP might be an alkylating agent. It could directly injure tumor DNA and cause tumor cell arrest at the G_0_/G_1_ phase in order to repair the damage. Our study using blocking agents indicated that Fas/Fas L and Bax were not cause tumor apoptosis molecules. The upper regulation of Fas and Bax protein was an association phenomenon.

RG2 cells have impaired p53 expression and homozygous deletions at the p16/Cdkn2a/Ink4a gene locus. Therefore, BP-induced apoptosis should be caused via a p53-indepedent pathway (Fas-induced apoptosis pathway) but not a p53-depedent pathway. However, although RG2 cells were impaired for p53 and p16, p27 and procaspase 9 expression induced by BP were increased in these cells. Thus, certain mechanisms of p53-independent activation of procaspase 9 and p27 induction may be involved in BP-induced cell apoptosis and cell growth inhibition. The molecular profile shown in Fig. (2) between DBTRG8401 and DBTRG-05 is much similar to RG2. The RG2 cells had no p53 protein expression. It might be the possibility of different profile of caspase-9 and p27 activation between DBTRG and RG2 cells.

Transdermally-applied BP quickly permeates the peripheral circulation system without accumulating in the skin; it is then distributed into the lung, liver, bile, brain and kidney. Total radioactivity, however, is decreased due to its excretion into the urine. In the case of intravenous administration, 80% of the administered BP is excreted into the urine within 24 h, while only 5% is excreted into the feces. The metabolite in urine is a cysteine conjugate (Sekiya *et al*. 2000). In our experiment, DBTRG-05MG cells (2.5 × 10^6^) were injected s.c. into the backs of nude mice (five to six/group) and treated with BP on days 4, 5, 6, 7 and 8 after tumor cell implantation. Survival of nude mice was significantly prolonged in the BP-treated groups, relative to the control group (Fig. 5b; *p* < 0.001). On day 200, the survival rate in the BP-800 mg/kg-treated group was 83.3% (5/6). Because BP is excreted into the urine within 24 h, it is interesting to note that five continuous doses showed significant tumor inhibition.

In conclusion, BP has potent anti-cancer effects on cell-cycle arrest and apoptosis. The present results provide new hope for chemotherapy of malignant brain cancer. This may lead to new therapeutic options in the treatment of malignant brain cancer, and an improved understanding of the interactions between phytochemicals with the genes that are critical to the regulation of brain cancer cells.

## Abbreviations used

AS: *Angelica sinensis*
AS-C: *A. sinensis* chloroform extract
BCNU: carmustine
BP: *n*-butylidenephthalide
CKI: Cyclin Kinase Inhibitor
DMSO: dimethylsulfoxide
MRI: magnetic resonance imaging
PI: propidium iodide

## References

[b1] Ye Y N, Koo M W, Li Y, Matsui H, Cho C H (2001a). *Angelica sinensis* modulates migration and proliferation of gastric epithelial cells. Life Sci.

[b2] Ye Y N, Liu E S, Li Y (2001b). Protective effect of polysaccharides-enriched fraction from *Angelica sinensis* on hepatic injury. Life Sci.

[b3] Graham D I, Lantos P L (2002). Greenfield's Neuropathology.

[b4] Santarius T, Kirsch M, Rossi M L, Black P M (1997). Molecular aspects of neuro-oncology. ClinNeurolNeurosurg.

[b5] Giese A, Bjerkvig R, Berens M E, Westphal M (2003). Cost of migration: invasion of malignant gliomas and implications for treatment. JClinOncol.

[b6] Blacklock J B, Wright D C, Dedrick R L (1986). Drug streaming during intra-arterial chemotherapy. JNeurosurg.

[b7] Shapiro W R, Green S B (1987). Reevaluating the efficacy of intra-arterial BCNU. JNeurosurg.

[b8] Elliott P J, Hayward N J, Huff M R, Nagle T L, Black K L, Bartus R T (1996). Unlocking the blood–brain barrier: a role for RMP-7 in brain tumor therapy. ExpNeurol.

[b9] Bello L, Carrabba G, Giussani C (2001). Low-dose chemotherapy combined with an antiangiogenic drug reduces human glioma growth *in vivo*. Cancer Res.

[b10] Cheng Y L, Chang W L, Lee S C (2004). Acetone extract of *Angelica sinensis* inhibits proliferation of human cancer cells via inducing cell cycle arrest and apoptosis. Life Sci.

[b11] Yim T K, Wu W K, Pak W F, Mak D H, Liang S M, Ko K M (2002). Myocardial protection against ischaemia-reperfusion injury by a *Polygonum multiflorum* extract supplemented ‘Dang-Gui decoction for enriching blood’, a compound formulation, *ex vivo*. PhytotherRes.

[b12] Abebe W (2002). Herbal medication: potential for adverse interactions with analgesic drugs. JClinPharmTher.

[b13] Tsai N M, Lin S Z, Lee C C, Chen S P, Su H C, Chang W L, Harn H J (2005). The antitumor effects of *Angelica sinensis* on malignant brain tumors *in vitro* and *in vivo*. ClinCancer Res.

[b14] Wang H, Chen R, Xu H (1998). Chemical constituents of radix *Angelicae sinensis*. Zhongguo Zhong Yao Za Zhi.

[b15] Teng C M, Chen W Y, Ko W C, Ouyang C H (1987). Antiplatelet effect of n-butylidenephthalide. BiochimBiophysActa.

[b16] Ko W C, Sheu J R, Tzeng S H, Chen C M (1998). The selective antianginal effect without changing blood pressure of n-butylidenephthalide in conscious rats. Planta Med.

[b17] Ko W C, Liao C C, Shih C H, Lei C B, Chen C M (2002). Relaxant effects of n-butylidenephthalide in isolated dog blood vessels. Planta Med.

[b18] Mimura Y, Kobayashi S, Naitoh T, Kimura I, Kimura M (1995). The structure-activity relationship between synthetic n-butylidenephthalide derivatives regarding the competence and progression of inhibition in primary cultures proliferation of mouse aorta smooth muscle cells. BiolPharmBull.

[b19] Sherr C J, Robert J M (1995). Inhibitors of mmmmalian G1 cyclin-dependent kinases. Genes Dev.

[b20] Reed S I, Bailly E, Dulic V, Hangst L, Resnitzky D, Slingerland J (1994). G1 control in mammalian cells. JCell Sci.

[b21] Dobashi Y, Takehana T, Ooi A (2003). Perspectives on cancer therapy: cell cycle blockers and perturbators. CurrMedChem.

[b22] Bardon S, Foussard V, Fournel S, Loubat A (2002). Monoterpenes inhibit proliferation of human colon cancer cells by modulating cell cycle-related protein expression. Cancer Lett.

[b23] Fukuoka K, Yamagishi T, Ichihara T (2000). Mechanism of action of aragusterol a (YTA0040), a potent antitumor marine steroid targeting the G(1) phase of the cell cycle. IntJCancer.

[b24] Ko W C (1980). A newly isolated antispasmodic-n-butylidenephthalide. Jpn JPharmacol.

[b25] Tano K, Dunn W C, Darroudi F, Shiota S, Preston R J, Natarajan A T, Mitra S (1997). Amplification of the DNA repair gene O^6^-methyguanine DNA methyltranferase associated with resistance to alkylating drugs in a mammalian cell line. JBiolChem.

[b26] Sekiya K, Tezuka Y, Tanaka K (2000). Distribution, metabolism and excretion of n-butylidenephthalide of Ligustici chuanxiong rhizoma in hairless mouse after dermal application. JEthnopharmacol.

[b27] Mahaley S J, Mettlin C, Natarajan N, Laws R J, Peace B B (1989). National survey of patterns of care for brain-tumor patients. JNeurosurg.

